# *Salmonella*’s lost phenotype: implications of sequence-based serotyping on the characterization of lipopolysaccharide-deficient *Salmonella* isolates

**DOI:** 10.1128/spectrum.02498-25

**Published:** 2026-01-27

**Authors:** Jasmin Wenderlein, Andreas J. Stroehlein, Michael Pietsch, Sandra Simon, Istvan Szabo, Burkhard Malorny, Marina C. Lamparter, Jennie Fischer

**Affiliations:** 1 Department Biological Safety, German Federal Institute for Risk Assessment 27652, Berlin, Germany; 2 Unit of Enteropathogenic Bacteria and Legionella-National Reference Centre for Salmonella and Other Bacterial Enteric Pathogens, Robert Koch Institute 235859 , Wernigerode, Germany; Virginia Polytechnic Institute and State University, Blacksburg, Virginia, USA

**Keywords:** *Salmonella*, whole-genome sequencing, lipopolysaccharide, serotyping, rough

## Abstract

*Salmonella* serotyping is shifting from slide agglutination toward whole-genome sequencing (WGS). While WGS allows for comprehensive analyses, phenotypic information about lipopolysaccharide-deficient (“LPS-rough”) isolates obtained from slide agglutination is lost. This discrepancy represents a challenge for *Salmonella* control in livestock because in the European Union, LPS-rough *Salmonella* isolated from food-producing animals that are untypable using slide agglutination alone are not subject to control measures, whereas isolates of certain serovars in certain matrices would be, when based on geno-serotyping. Here, we provide an account of the relevance of this phenotype in the context of routine diagnostics and food safety by characterizing the occurrence, diversity, and isolation matrices of LPS-rough isolates among non-human *Salmonella enterica* subsp. *enterica* isolates from Germany. Using available WGS data, we examined phylogenetic relationships and associations with certain genomic features. On average, 5% of isolates exhibited an LPS-rough phenotype across 46 serovars, but clonal distribution of this phenotype along the food chain was not evident. LPS-rough isolates were more commonly found in *S*. Choleraesuis isolated from wild boar and in *S*. Typhimurium isolated from pork products, compared with other matrices. We also found associations with two virulence factors, an AMR gene, and a plasmid marker. The present work lays the foundation for future research into the role of certain matrices, environments, or genomic factors in the development of LPS-rough *Salmonella* isolates and will facilitate the elucidation of the genomic basis of this phenotype, which may improve recommendations regarding risk management and control measures.

**IMPORTANCE:**

The present work highlights some of the challenges associated with the recent shift from serology- to sequence-based typing of *Salmonella enterica* serovars and provides a national perspective on the presence and relevance of the lipopolysaccharide-deficient (“LPS-rough”) phenotype in samples obtained from food, animals, and the environment, including considerations regarding the choice of typing method. We provide evidence that clonal distribution of isolates with this phenotype is unlikely, but that certain environments may favor its development, and certain genomic factors may increase survival rates of LPS-rough isolates when exposed to environmental stressors. These findings could have important implications for regulations regarding the surveillance and management of *Salmonella* isolated from food, feed, and animals in the future, in particular in the context of using different typing methods, and warrant further detailed research.

## INTRODUCTION

The methodology for identifying *Salmonella* serovars in both routine diagnostics and research applications is currently shifting from classical slide agglutination toward geno-serotyping using whole-genome sequencing (WGS). In addition to predicting the serotype (1, 2), WGS can accelerate the investigation of outbreaks (3–5) and enables typing and characterization of antimicrobial resistance (AMR) genes (6–8), virulence genes (9–11), and mobile genetic elements (12–14). Accordingly, many countries have started transitioning from slide agglutination using the White-Kauffmann-Le Minor scheme (15) to WGS-based geno-serotyping. Nonetheless, slide agglutination is still defined as the gold standard by European regulations (i.e., CR [EC] 1237/2007, CR [EU] 517/2011, CR [EU] 200/2012, CR [EU] 1190/2012, R [EC] 2160/2003, and CR [EC] 2073/2005). This shift in methodology represents a challenge since neither of the two methods achieves a complete characterization of pheno- and genotype for isolates that are lipopolysaccharide-deficient (“LPS-rough”) (16). On the one hand, slide agglutination can identify phenotypically LPS-rough *Salmonella* isolates but cannot determine a serotype due to unspecific agglutination with antisera against multiple O-antigen groups caused by a defect in LPS assembly. On the other hand, geno-serotyping of LPS-rough isolates is generally possible using WGS data, but information about the LPS-rough phenotype is lost in the absence of slide agglutination results. Given these limitations, the choice of method is of importance, as it can lead to different typing results and, thus, can have an effect on the applied control measures.

The European regulations relating to *Salmonella* spp. control in poultry (CR [EC] 1237/2007, CR [EU] 517/2011, CR [EU] 200/2012, CR [EU] 1190/2012, and R [EU] 2160/2003) and the German regulation for *Salmonella* in poultry (*Geflügel-Salmonellen-Verordnung*) define certain *Salmonella enterica* subsp. *enterica* (*Salmonella* or *S.*) serovars of categories I (*S*. Enteritidis, *S*. Typhimurium) and II (*S*. Hadar, *S*. Virchow, and *S*. Infantis) that are subject to control measures in Europe and Germany. Since LPS-rough *Salmonella* isolates cannot be assigned to category I or II serovars by slide agglutination-based serotyping, they are excluded from such measures. In contrast, WGS-based methods are generally able to geno-serotype LPS-rough isolates. So far, it is unclear whether LPS-rough strains typed as category I or II serovars by WGS alone should be subject to control measures, given that there is still a lack of understanding about the virulence of LPS-rough *Salmonella,* with some studies reporting equal (17, 18) and others suggesting reduced virulence (19–21). LPS-rough phenotypes range from ‘deep-rough’ (i.e., isolates that have a truncated core or a complete core with no O-antigen [22–24]) to ‘semi-rough’ (i.e., isolates that have a complete core and parts of the first repeat unit of the O-antigen or one complete repeat unit of the O-antigen [22, 23]). In *Salmonella* spp., LPS biosynthesis is governed by a large and diverse set of genes (25, 26), whereby single gene defects can have pronounced positive or negative effects on virulence, such as motility (21), attachment to epithelial cells (27), host cell invasion capability (21, 27–29), establishment in the cells or organism, and immune evasion (30, 31). Current, limited evidence suggests that mutations leading to a ‘deep-rough’ phenotype reduce virulence more than mutations leading to a ‘semi-rough’ phenotype (17). However, a comprehensive understanding of the underlying genomic basis of these variations is lacking, given that most studies investigating LPS-rough *Salmonella* spp. were conducted before WGS became commonplace. Based on currently limited data on virulence, the detection of an LPS-rough *Salmonella* spp. isolate alone may, therefore, not be sufficient for appropriate decision-making in a food safety context. Additional, comprehensive whole-genome data are now needed to facilitate a more refined characterization of the genomic basis of this phenotype.

To better assess the relevance of LPS-rough *Salmonella* spp. in the context of routine diagnostics and food safety, a comprehensive documentation of their occurrence, genetic diversity among different serovars, isolation matrices (food, environment, livestock, and wildlife), and geographic origins is needed. Here, we begin this process by analyzing isolates collected at the German National Reference Laboratory (NRL) for *Salmonella* between 2019 and 2023 for which phenotypic information, geographic location, and isolation matrix metadata were available. Additionally, for isolates for which WGS data were available, we determined their phylogenetic relationships and other genomic features, including the presence of plasmids, as well as AMR and virulence genes. This study provides an improved perspective on the occurrence and distribution of LPS-rough *Salmonella* isolates in Germany and establishes a solid data foundation for further research, which should help inform future recommendations regarding risk management and control.

## MATERIALS AND METHODS

### Selection of isolates and associated sequence data

To assess the occurrence of LPS-intact (”-smooth”) and -deficient (“-rough”) *Salmonella* isolates, we selected a cross-sectional data set from the strain collection at the NRL for *Salmonella* submitted between 2019 and 2023. This selection represented both opportunistic and focused sampling efforts across all 16 federal states in Germany and consisted of non-human isolates with serovars of high public health relevance in Germany, which were collected from diverse animal species (including wildlife), food, feed, the environment, or unspecified sources. Isolates from national sampling programs, regional monitoring studies, and routine diagnostics were included, and multi-sampling duplicates were removed. At the NRL for *Salmonella*, a selection of isolates is routinely subjected to WGS. Isolates are selected for sequencing based on national monitoring and control programs, current outbreaks, or research projects and include LPS-rough *Salmonella* isolates. Isolate-associated Illumina short-read libraries are constructed using the Nextera DNA Flex Library Preparation Kit and are sequenced on Illumina NextSeq, MiSeq, or iSeq instruments (32). All sequence data at the NRL are routinely processed, quality-checked, and assembled using the AQUAMIS pipeline (v1.4.0 [33]).

### Serotyping

As part of the routine diagnostic procedure at the NRL for *Salmonella*, isolates of this study underwent slide agglutination according to the White-Kauffmann-Le Minor scheme (15) using O- and H-antigen specific sera (Sifin Diagnostics, Berlin, Germany and SSI Diagnostica A/S, Hillerod, Denmark). Subsequent biochemical testing of isolates was carried out according to White-Kauffmann-Le Minor (15) and CEN ISO/TR 6579-3:2014 (34) for subspecies identification or to subtype isolates (e.g., *S*. Choleraesuis). Isolates that showed a specific reaction to only one O-antigen group were defined as phenotypically LPS-smooth, and isolates that showed an unspecific reaction with multiple O-antigen groups were defined as phenotypically LPS-rough (34). Isolates that showed an LPS-rough phenotype by slide agglutination were subjected to a *Salmonella*-specific PCR to confirm the genus (35).

### Genoserotyping and annotation of genomic features

For isolates sequenced as part of the routine WGS workflow at the NRL for *Salmonella* (33, 36), the BakCharak pipeline (https://gitlab.com/bfr_bioinformatics/bakcharak; v.3.0.4) was employed to determine geno-serotype, multi-locus sequence type (MLST), antimicrobial resistance (AMR) genes, virulence factors, and plasmids from genome assemblies using the programs *SISTR* v.1.1.1 (37), *mlst* v.2.22.0 (https://github.com/tseemann/mlst) (38), *AMRfinder* v.3.10.45 (*ncbi-amrfinder* database 2022-10-11.2) (39), and *abricate* v.1.0.1 (https://github.com/tseemann/abricate) with the *VFDB* (version 2022-08-26) (40) and *plasmidfinder* (version 2022-07-13) (12, 41) databases, respectively.

### Phylogenetic relationship of LPS-rough and -smooth *Salmonella* isolates

To investigate phylogenetic links between isolates and assess whether there are established *Salmonella* clones with an LPS-rough phenotype, genetic relationships of all isolates over the entire sampling period were compared per serovar. Core-genome MLST (cgMLST) allelic distances (ADs) were calculated at cluster thresholds (CTs) of 20, 10, and 5 AD using *chewieSnake* v.3.2 (42), employing *chewBBACA* v.2.0.16 (43) with a modified *EnteroBase* cgMLST scheme (3,000 loci, [44]). To reflect a range of CTs commonly applied in outbreak cluster definition for *Salmonella enterica* (cf. [45–47]), CTs of 20, 10, and 5 AD were chosen. Single-linkage clustering of the distance matrices was carried out per serovar using the *hclust* method in *R* v.4.3.1 (48).

### Identification of relationships between phenotypic, genomic, and epidemiological isolate characteristics

To identify possible genomic or epidemiological characteristics that relate to an LPS-rough phenotype, we investigated associated isolation matrix metadata and genomic features (plasmids, resistance and virulence genes, MLST, and cgMLST cluster) for all isolates of each of the six major serovars *S*. Typhimurium, *S*. Enteritidis, *S*. Infantis, *S*. Choleraesuis, *S*. Derby, and *S*. Paratyphi B *d*-tartrate-positive (hereafter referred to as *S*. Paratyphi B var. Java). Genomic characteristics and matrix metadata were binary-encoded as present or absent, and the proportions of isolates having each feature were calculated. We then tested, for each feature, whether a significantly larger proportion of isolates with this feature was LPS-rough (H1; Fisher’s exact test [49], *P* ≤ 0.05, correction for multiple testing using the Bonferroni method [50]) compared with the overall proportion of LPS-rough isolates per serovar. Non-independent (i.e., significant) metadata features or genomic characteristics were considered potential associations and investigated further.

## RESULTS

### Abundance and characteristics of phenotypically LPS-rough isolates from the German NRL strain collection

**Fig 1:**
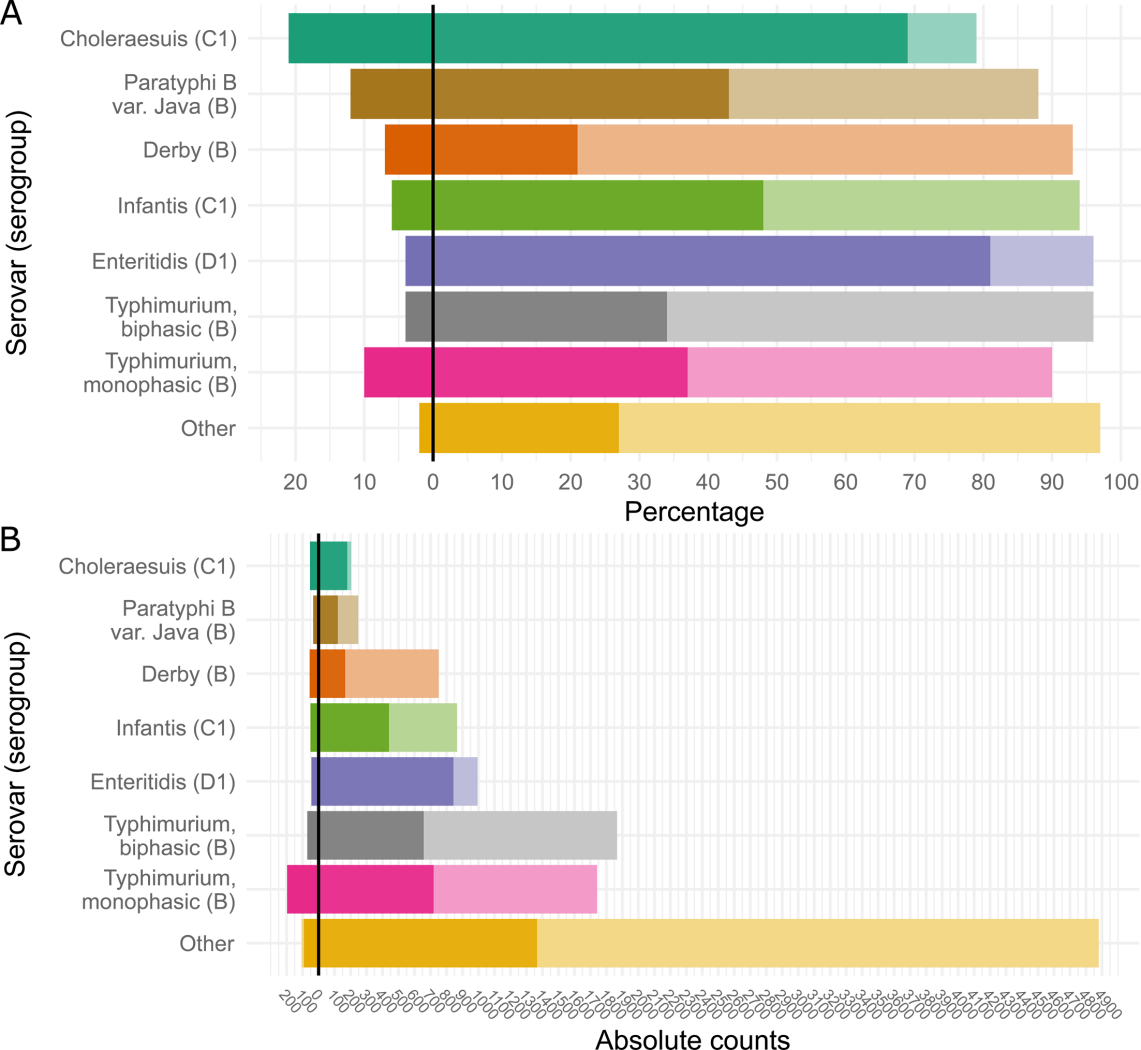
Number of isolates for the most common serovars and the fraction of those that have an LPS-rough phenotype (bar chart to the left of 0) and those that have an LPS-smooth phenotype (bar chart to the right of 0). The fraction of samples that have been sequenced using WGS is represented in a dark hue, and the fraction of samples that have not been sequenced is shown in a lighter hue. Relative (**A**) and absolute abundances (**B**) are shown. Isolates with less common serovars or those that could not be serologically typed and had not been sequenced are summarized as “other.”

**Fig 2:**
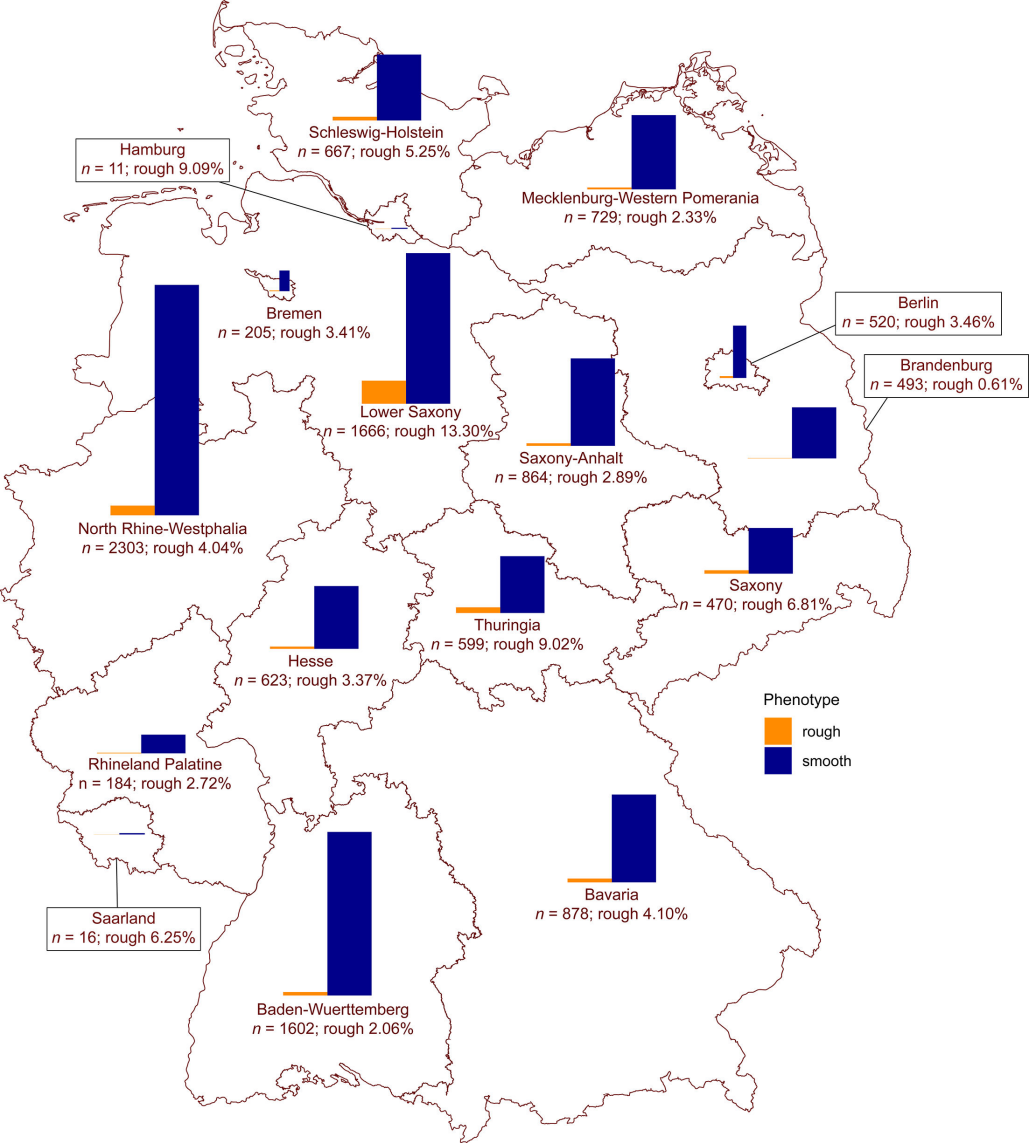
Overview map of LPS-rough (orange) and -smooth (blue) isolates sampled between 2019 and 2023 in individual federal states of Germany. Absolute numbers include sequenced and un-sequenced isolates. Created with mapchart.net.

**Fig 3:**
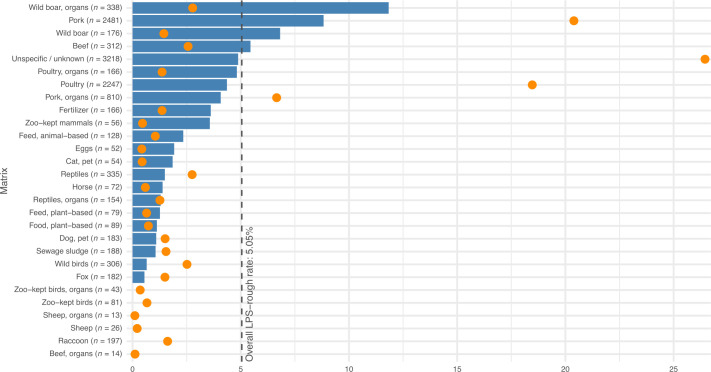
Sampling breakdown by isolation matrices. Orange dots indicate the relative contribution of individual isolation matrices to the entire data set. Blue bars represent the rate of LPS-rough isolates for each individual isolation matrix. A dotted line indicates the overall rate of LPS-rough isolates, irrespective of the isolation matrix.

**Fig 4:**
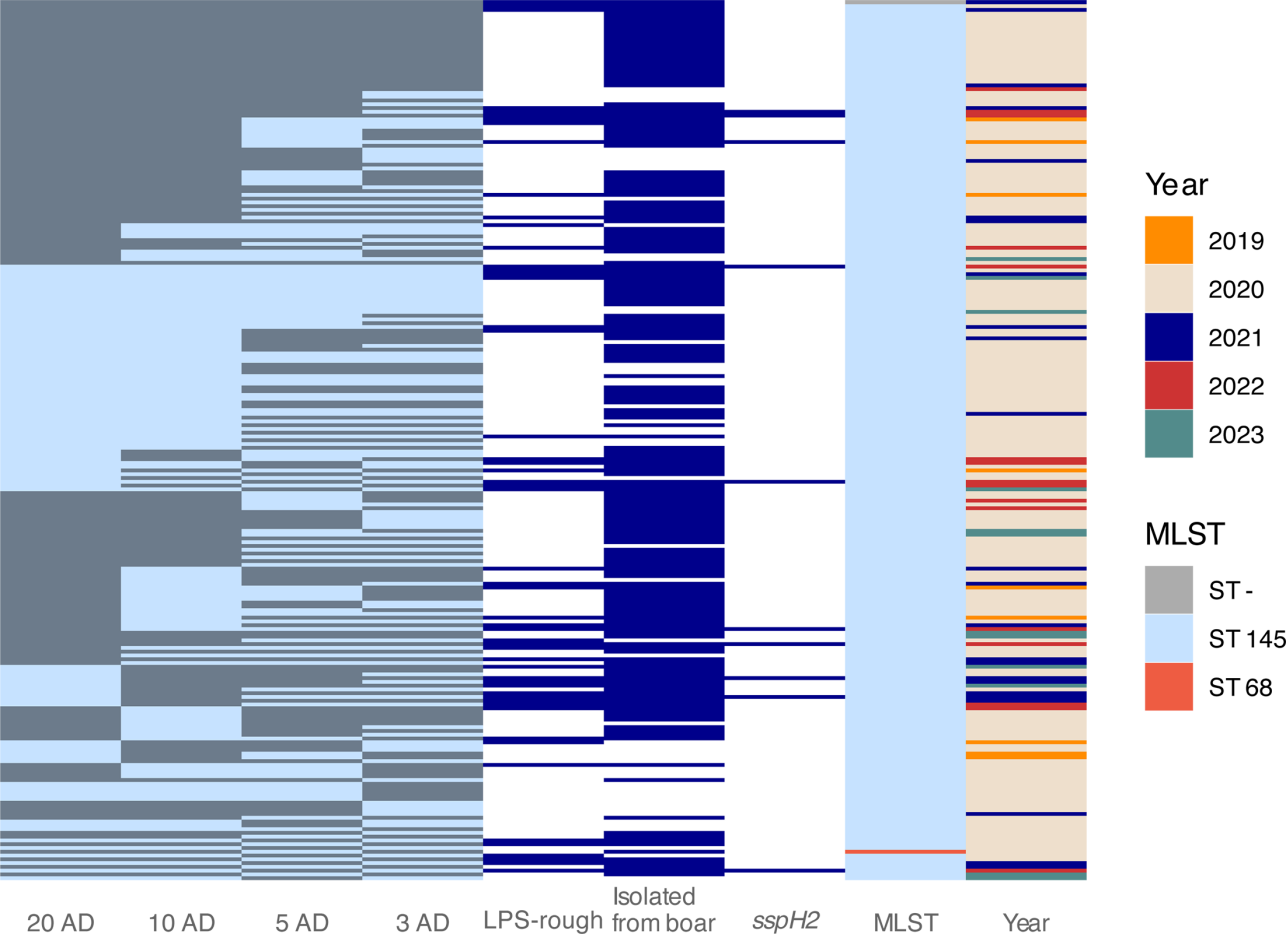
Phylogenetic relationships of sequenced *S.* Choleraesuis isolates (*n* = 233) and associated phenotypic, genomic, and sampling metadata. Hierarchical clustering based on core-genome multi-locus sequence typing (cgMLST) is shown for cluster thresholds of 20, 10, 5, and 3 allele distances (ADs) (clusters indicated by alternating gray and light blue blocks). Metadata features that were statistically overrepresented (*P* ≤ 0.05) among LPS-rough *S.* Choleraesuis isolates, as well as multi-locus sequence type (MLST) and sampling year are shown.

A total of 12,166 isolates were selected from the NRL for *Salmonella* strain collection, of which 5,090 (41.8%) had been sequenced (Data S1). The percentage of isolates that had been sequenced varied per serovar (Fig. 1A), with the least isolates sequenced for *S*. Derby (27.5%) and the most sequenced for *S*. Choleraesuis (90.3%). The sampled isolates also varied in terms of sender states and isolation matrices (Fig. 2 and 3). The most common isolation matrices overall were pork and pork-derived products (most common for *S*. Derby and *S*. Typhimurium) and poultry (most common for *S*. Enteritidis, *S*. Infantis, and *S*. Paratyphi B var. Java). For *S*. Choleraesuis, the most common isolation matrix was wild boar (Fig. 4; Fig. S1). The number of sampled isolates per year was relatively uniform for each serovar and isolation matrix (Fig. S1). Of all selected isolates, 614 (5.0%) exhibited an LPS-rough phenotype; for most of these isolates (600; 97.7%), WGS data were available. The five most common serovars in this data set determined using slide agglutination included *S*. Typhimurium (*n* = 1,821) and its monophasic variant (antigen formula “4,[5],12:i:-“; *n* = 1,769), *S*. Enteritidis (*n* = 1,000), *S*. Infantis (*n* = 837), *S*. Derby (*n* = 749), and *S*. Paratyphi B var. Java (*n* = 231). The majority (73.3%; *n* = 4,219) of the remaining 5,759 isolates was assigned to one of 249 other, less common serovars (Data S1). For the remaining 26.3% of isolates (*n* = 1,540), no serovar could be determined using slide agglutination (i.e., they were assigned to one of the categories “subspecies I,” “*Salmonella* group E,” “*Salmonella* spp.,” or “rough phenotype”). For those of them that had been sequenced (*n* = 957, including 600 LPS-rough isolates), one of 65 distinct serovars was inferred by geno-serotyping. The combination of slide agglutination and genomic data enabled typing of a total of 1,938 *S*. Typhimurium isolates, 1,939 of its monophasic variant, as well as 1,039 *S*. Enteritidis, 917 *S*. Infantis, 807 *S*. Derby, 282 *S*. Paratyphi B var. Java, and 258 *S*. Choleraesuis isolates. Isolates with an LPS-rough phenotype were found across 46 serovars (Data S1), with monophasic *S*. Typhimurium (*n* = 197) and biphasic *S*. Typhimurium (*n* = 71) together representing 43.6% of all LPS-rough isolates. The six most common serovars comprised between 5.5 and 32.1% of all LPS-rough isolates detected. The serovar with the highest occurrence of LPS-rough isolates relative to the number of collected isolates was *S*. Choleraesuis: of 258 isolates, 54 were LPS-rough (20.9%). In comparison, serovars with highest public health relevance in Germany (i.e., monophasic *S*. Typhimurium, *S*. Infantis, *S*. Enteritidis, and biphasic *S*. Typhimurium) had relative abundances of 10.2, 5.6, 4.3, and 3.7%, respectively (Fig. 1A).

### Phylogenetic relationships of LPS-rough and -smooth isolates per serovar

**Fig 5:**
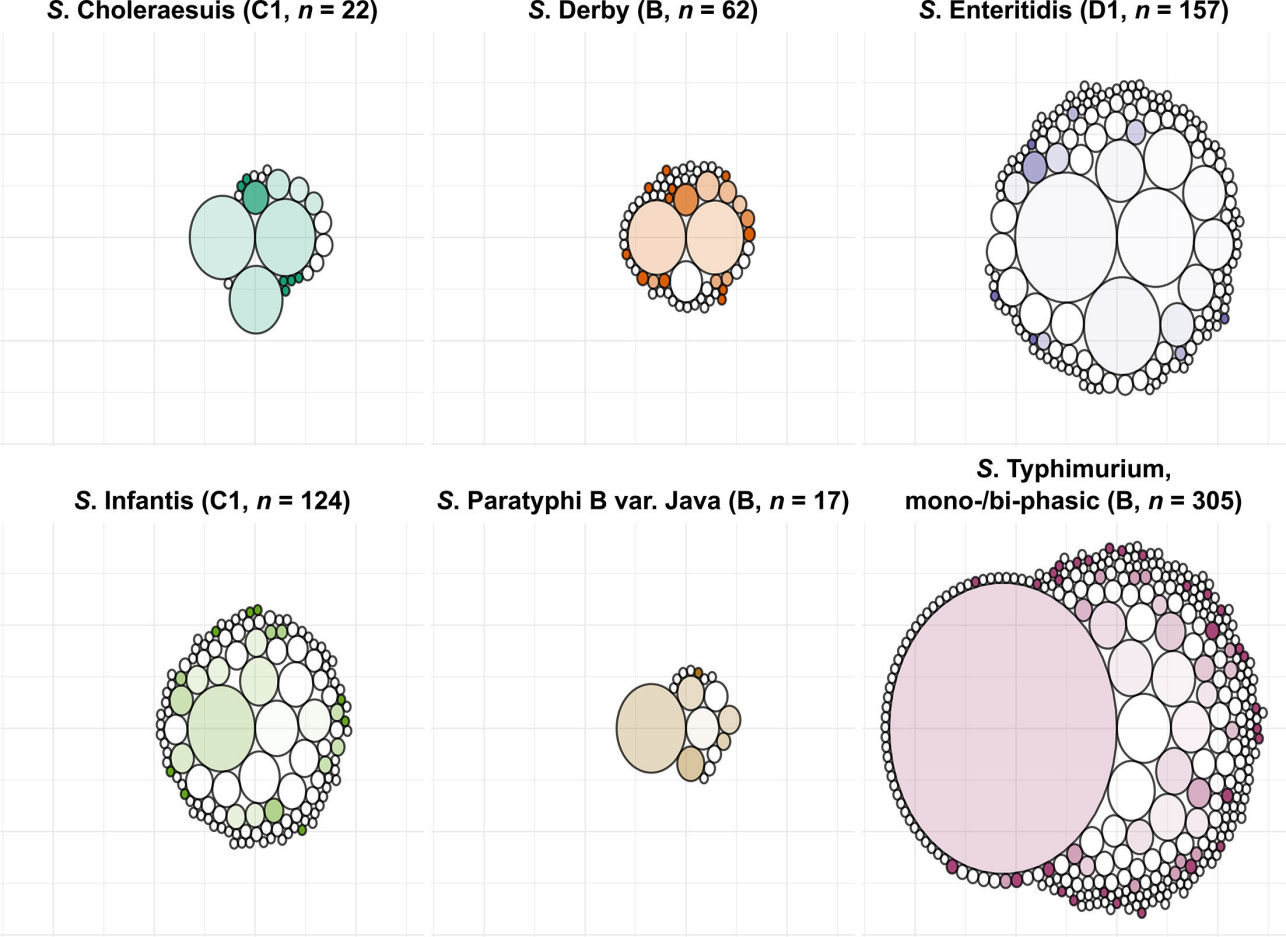
Number and size of 20 allele distance (AD) core-genome multi-locus sequence typing (cgMLST) clusters and singletons per serovar (serogroup in brackets) for the six most common serovars. Each circle represents a cluster or singleton, and the size of the circle is proportional to the number of isolates in each cluster. The color intensity represents the number of LPS-rough isolates in each cluster, with more opaque clusters having a higher proportion of LPS-rough isolates.

**Fig 6:**
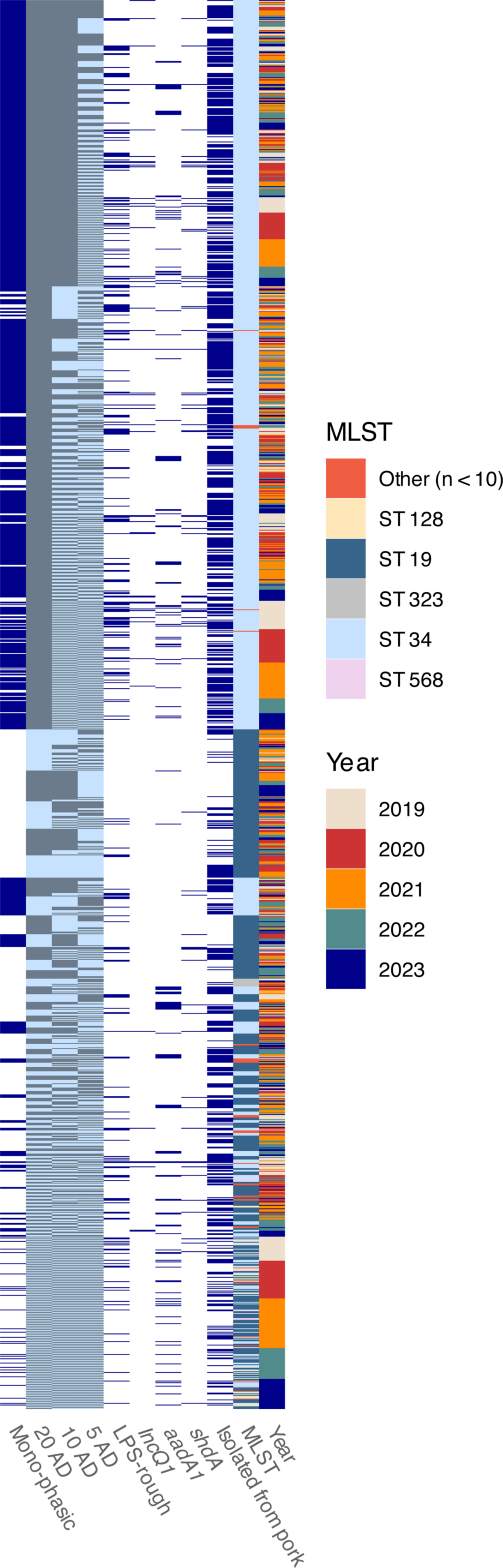
Phylogenetic relationships of sequenced *S.* Typhimurium isolates (*n* = 1,644; including mono- and biphasic isolates, monophasic isolates indicated in the first column) and associated phenotypic, genomic, and sampling metadata. Hierarchical clustering based on core-genome multi-locus sequence typing (cgMLST) is shown for cluster thresholds of 20, 10, and 5 allele distances (ADs) (clusters indicated by alternating gray and light blue blocks). Metadata features that were statistically overrepresented (*P* ≤ 0.05) among LPS-rough *S.* Typhimurium isolates (including a plasmid marker, AMR gene, virulence factor and isolation matrix), as well as multi-locus sequence type (MLST) and sampling year are shown. Minor MLSTs (*n* < 10) are collapsed into the category “other.”

Next, we investigated the genomic relatedness of all sequenced LPS-rough and -smooth isolates that belonged to the six most common serovars (*n* = 3,632). These isolates were clustered separately per serovar based on their cgMLST profiles to assess whether specific, stable clones with an LPS-rough phenotype are consistently circulating in Germany. At a CT of 20 AD, a total of 258 clusters were defined, with the fewest clusters detected for *S*. Paratyphi B var. Java (*n* = 9) and the most for *S*. Typhimurium (monophasic and biphasic combined) (*n* = 104; Table 1; Fig. 5). Between 4.7 (*S*. Choleraesuis) and 19.8% (*S*. Derby) of isolates were not assigned to a cluster (i.e., they were singletons). For each serovar, there was a small number of large clusters (maximum cluster size between 56 and 851), including an *S*. Typhimurium cluster (cluster 2) containing more than half of all isolates for that serovar (Fig. 5 and 6). There were no clusters that solely contained LPS-rough isolates, except for small clusters of *S*. Derby (three clusters out of 62 clusters, two isolates each) and *S*. Typhimurium (six clusters, two to three isolates each). For *S*. Enteritidis, *S*. Infantis, and *S*. Typhimurium, most clusters (66.0–74.6%) solely contained smooth isolates, whereas for *S*. Choleraesuis, *S*. Derby, and *S*. Paratyphi B var. Java, this proportion was lower (27.8–36.4%). To ensure that none of the 20 AD clusters contained phylogenetically linked sub-clusters of LPS-rough isolates, we investigated all 20 AD clusters that contained at least two LPS-rough isolates (*n* = 26) at a CT of 10 AD. This revealed 178 individual clusters, of which 102 (cluster size 2–20) contained no LPS-rough isolates. Among the remainder, 15 (8.4%) very small clusters entirely consisted of two (*n* = 9), three (*n* = 5), or four (*n* = 1) LPS-rough isolates. The four isolates in the latter cluster (*S*. Typhimurium cluster 68; Data S1) originated from various pork products and were submitted by two different senders from different federal states between 2019 and 2020. At the 5 AD level, they clustered into three separate clusters (clusters 91, 151, and 195; Data S1). Since no sizable (*n* ≥ 5) clusters solely containing LPS-rough isolates were found, we next assessed whether clusters existed that contained a significantly (*P* ≤ 0.05) higher number of LPS-rough isolates than what would be expected based on the overall abundance per serovar. We identified two clusters: cluster 1 (cluster size: 75) for *S*. Infantis contained 18 LPS-rough isolates (24.0%) compared with an overall rate of sequenced *S*. Infantis LPS-rough isolates of 10.4% (fold change 2.31×); and cluster 10 for *S*. Enteritidis (cluster size: 10) contained six LPS-rough isolates (60.0%) compared with an overall rate of sequenced *S*. Enteritidis LPS-rough isolates of 5.07% (fold change: 11.8×). Neither of these two clusters was significantly associated with a specific isolation matrix, sampling year, or MLST type (Fig. S2).

**TABLE 1:** Key metrics of cgMLST clusters (20 AD cluster threshold) and the proportions of LPS-rough and -smooth isolates within them

Serovar	Number of isolates	Number of 20 AD clusters	Number of singletons	Number of LPS-rough singletons	Number of LPS-smooth singletons	Fraction of singletons	Fraction of rough singletons	Maximum cluster size	Proportion of 100%-LPS-rough clusters	Proportion of 100%-LPS-smooth clusters
Choleraesuis	233	11	11	6	5	4.7	54.6	70	0	36.4
Derby	222	18	44	8	36	19.8	18.2	56	16.7	27.8
Enteritidis	888	63	94	4	90	10.6	4.3	170	0	74.6
Infantis	491	53	71	8	63	14.5	11.3	75	0	66.0
Paratyphi B var. Java	154	9	8	1	7	5.2	12.5	79	0	33.3
Typhimurium (mono- and biphasic)	1644	104	201	24	177	12.2	11.9	851	5.8	67.3

### Association of LPS-rough isolates with genomic characteristics or with isolation matrices

Next, we investigated a total of 362 characteristics (“features”) for potential associations with the LPS-rough phenotype, including 171 virulence factors, 96 AMR genes/gene profiles, 65 plasmid markers, and 30 isolation matrices. Although none of these characteristics were uniquely identified in LPS-rough isolates only, we found a significant overrepresentation (*P* ≤ 0.05; Table 2; Data S2) of LPS-rough isolates among isolates with the virulence factor *shdA* (fold change between 4.2× and 8.6×) for *S*. Enteritidis (Fig. S2B) and *S*. Typhimurium (both mono- and biphasic; Fig. 6). LPS-rough isolates were also overrepresented among *S*. Choleraesuis isolates that encoded the *sspH2* virulence factor (100% LPS-rough; Fig. 4) compared with the LPS-rough rate for *S*. Choleraesuis overall (23.2%, fold change: 4.31×). Our assessment also revealed an overrepresentation (fold change: 2.6×) of LPS-rough isolates among *S*. Typhimurium isolates that encoded the AMR gene *aadA1*. Additionally, monophasic *S*. Typhimurium LPS-rough isolates were associated with the *IncQ* incompatibility (Inc) plasmid family; the *IncQ1_1* marker was found in 49 monophasic *S*. Typhimurium isolates, and of those, 43 (87.8%) were LPS-rough compared with 21.5% of sequenced isolates being LPS-rough overall for this serovar (fold change: 4.08×; Fig. 3). Of those 43, 38 (88.4%) belonged to the same 20 AD cgMLST cluster (cluster 2, *n* = 851; Fig. 6).

**TABLE 2:** Summary of genomic features, cgMLST clusters, and isolation matrices significantly (*P* ≤ 0.05) associated with the LPS-rough phenotype

Serovar	Genomic features significantly associated with LPS-rough phenotype	cgMLST clusters (20 AD) significantly associated with LPS-rough phenotype	Isolation matrices significantly associated with LPS-rough isolates	Range of LPS-rough fractions in 20 AD clusters (≥5)	Fraction of LPS-rough isolates among sequenced isolates
Choleraesuis	*sspH2* (virulence factor)	None	Wild boar (incl. organs)	0–72.7	22.1
Enteritidis	*shdA* (virulence factor)	Cluster 10	None	0–60.0	5.6
Infantis	None	Cluster 1	None	0–50.0	10.8
Typhimurium (monophasic)	*shdA*, *IncQ1* (plasmid marker)	None	None	0–44.4	17.1
Typhimurium	*shdA*, *aadA1* (AMR gene)	None	Pork	0–44.4	17.1

In addition to associations with genomic features, two isolation matrices showed a significant overrepresentation of LPS-rough isolates (Table 2): in *S*. Typhimurium, LPS-rough isolates were 1.69× more common in isolates originating from pork (16.5% vs. 9.75% LPS-rough abundance, Fig. 3 and 6), and in *S*. Choleraesuis, LPS-rough isolates occurred 1.23× more often in isolates isolated from wild boar matrices (Fig. 3 and 4). The latter isolates were evenly distributed across four 20 AD cgMLST clusters (clusters 2, 3, 4, and 7; Data S1) which also contained between 27.3 and 81.4% of LPS-smooth isolates.

## DISCUSSION

In this work, we have screened a large data set of *Salmonella* isolates that were collected over a period of 5 years. These isolates reflect not only the diversity of *Salmonella* strains circulating in the German food production sector and in wildlife, but also opportunistic sampling efforts and the respective sampling foci of individual senders. Taking into account this limitation, our assessment of the distribution of LPS-rough *Salmonella* isolates does not support the presence of stable LPS-rough clusters (clones) in certain animal reservoirs or among specific food production lines that have persisted over several years at a CT of 10 AD, a relatively "relaxed" CT (compared with a CT of 3–5 AD frequently used in salmonellosis outbreak situations [45]). Instead, our findings indicate that a relatively conserved *S*. Typhimurium lineage has established in the pork production line (cf. [51], Fig. 6), in which LPS-rough isolates have emerged randomly. In this context, one LPS-rough strain isolated from pork was part of a recognized German *S*. Typhimurium human salmonellosis cluster, but the two most closely related isolates were LPS-smooth (data not shown). This example suggests that genomic characteristics causing LPS-rough phenotypes do not affect the inference of genomic relatedness among isolates using cgMLST; based on current evidence, alterations of single genes involved in LPS biosynthesis induce the LPS-rough phenotype (52), which would result in a single allelic difference, at most. Additionally, the *wzy* gene, mutations/truncations of which are considered one determinant of the LPS-rough phenotype (22), is not part of the commonly used cgMLST v2 (EnteroBase) scheme at all. Taken together, we want to emphasize the importance of including LPS-rough isolates (detected using slide agglutination) in WGS-based outbreak investigations to facilitate or improve source attribution because we consider it more likely that LPS-rough strains develop arbitrarily along established lineages. In the context of European *Salmonella* control programs, risk management decisions should be based on the identified geno-serotype, given that phenotypically LPS-rough isolates geno-serotyped as *Salmonella* category I or II likely reflect a population comprising both LPS-smooth and -rough variants, rather than one that is purely LPS-rough. This aspect is particularly relevant for settings where broad and untargeted sampling strategies are used, such as boot swabbing on poultry farms.

Although our work suggests that LPS-rough isolates mainly emerge sporadically, certain matrices or environments may favor the development of the LPS-rough phenotype through a higher rate of spontaneous mutations in genes involved in LPS synthesis. Based on the present findings, wild boar (*S*. Choleraesuis, cf. [32]) and pork products (*S*. Typhimurium) may represent such favorable environments for certain serovars. Similarly, LPS-rough isolates from human clinical samples (collected at the National Reference Center for *Salmonella* at the Robert Koch Institute, personal communication) are overrepresented in urine samples (38.1% of all LPS-rough isolates, relative to 4.5% of all *Salmonella* isolates originating from urine overall) despite a low overall proportion of LPS-rough isolates among all isolates received per year (1.8–2.4%). The higher number of LPS-rough isolates in human urine samples may be explained by a combination of environmental stressors associated with the human urinary tract (e.g., nutrient scarcity, an average pH of 6, uric acid, urea, and ammonia) that might drive the development of mutations leading to an LPS-rough phenotype.

Taken together, it is unclear if one, multiple, or all associations observed here lead to a higher frequency of spontaneous mutations that then cause an LPS-rough phenotype. It is also possible that one or more other, potentially serovar-specific genetic factors, which were not the subject of the present work, contribute to this phenotype. In this context, the role of specific food matrices and environmental stress, as well as the genetic diversity among LPS-rough strains, should be explored in more detail in the future. Irrespective of the driving factors behind this phenotype, the fact that LPS-rough *Salmonella* occur in outbreak clusters and that LPS-rough human clinical isolates have been reported raises the question of whether such isolates are—as some studies suggest—less virulent or equally virulent compared with LPS-smooth isolates (17–21). In this context, it is also of interest to which extent mixed populations of LPS-smooth and spontaneously developed LPS-rough bacteria co-exist in an infected individual. These aspects may affect the interpretation of whether LPS-rough isolates can cause infection.

In general, multiple distinct genetic factors within the LPS-encoding regions can confer an LPS-rough phenotype, which may have an impact on the competitive fitness of a strain and its ability to cause an infection in animals and humans. However, the established distinction between ‘deep-rough’ and ‘semi-rough’ strains does not consider all possible genetic defects in genes involved in LPS biosynthesis in *Salmonella* (25, 26). Importantly, routine slide agglutination-based determination of the LPS-rough phenotype does not reliably distinguish between any of the pheno- and genotypic subtypes despite their possible effects on strain virulence.

In addition to genes encoding components directly involved in LPS biosynthesis, resistance genes and other virulence factors may play an equally important role for the persistence and overall virulence of LPS-rough strains. For example, the overrepresentation of the virulence factors *shdA* (encoding an outer membrane fibronectin-binding protein) in LPS-rough *S*. Enteritidis (Fig. S2B) and *S*. Typhimurium (Fig. 6) and of *sspH2* (encoding a type three effector protein) in LPS-rough *S*. Choleraesuis (Fig. 4) suggests a possible role for these factors in compensating for shortcomings of LPS-rough strains with regard to fitness or ability to survive by increasing bacterial attachment (53) or subverting immune responses (54). Since most studies researching LPS-rough *Salmonella* were conducted before WGS was widely used, it is not clear if certain virulence factors were present in the strains from which LPS-rough mutants were constructed and whether the reported findings are, therefore, specific to certain serovars or even strains due to a specific genomic background. These aspects should be the subject of more detailed investigations in the future.

Overall, there is little evidence in the literature for a decreased virulence of LPS-rough *Salmonella* (17, 20, 27–30). However, one of the factors that has been described to reduce colonization by LPS-rough *Salmonella* may be the competition with other bacterial taxa that occupy similar nutrient niches (55, 56). The genomic elements identified here as more common (albeit not uniquely present) in LPS-rough isolates may indirectly provide LPS-rough *Salmonella* strains with an advantage in such competitive environments and may facilitate survival, or even colonization and infection. These aspects should be the subject of future genomic and functional investigations. In this context, the presence and relevance of LPS-rough *Salmonella* in human salmonellosis cases and outbreaks should be investigated in detail to better assess the health risk for consumers.

In the context of the association of the *IncQ1* plasmid family with the LPS-rough phenotype in monophasic *S*. Typhimurium (Fig. 6), a chromosomal integration of an *IncQ* plasmid *via* a Tn*21*- and IS*26*-mediated transposition into the *fljBA* operon and its flanking regions is well documented (57, 58); it gave rise to the European ST34 clone that carries the ASSuT (ampicillin, streptomycin, sulfonamides, tetracycline) resistance profile (51, 59). However, given that the rate of LPS-rough isolates within cgMLST cluster 2 did not differ from that of all sequenced monophasic *S*. Typhimurium isolates overall, we deem the establishment of a stable LPS-rough lineage unlikely and consider it more plausible that the mobile genetic elements that were integrated into the *fljBA* region of the chromosome in the monophasic ST34 clone may sporadically disrupt the gene clusters encoding enzymes for LPS biosynthesis (i.e., *waa*- and *wba*-gene clusters) through transposition events, thus causing an LPS-rough phenotype. This is to some extent supported by evidence showing that transposition sites are biased toward abrupt compositional shifts in target DNA (60), such as the shift from a relatively high-GC to a low-GC nucleotide composition in the gene clusters encoding enzymes for LPS biosynthesis (61). Accordingly, the *IncQ1* association probably reflects independent, but coinciding transposase-mediated mechanisms among different ST34 monophasic *S*. Typhimurium sub-lineages. A higher rate of contig breaks in the LPS region of short-read genome assemblies of LPS-rough isolates compared with LPS-smooth isolates (data not shown) lends some additional, preliminary support to this hypothesis, given that the presence of multiple identical insertion sequences in the genome represents a challenge for short-read genome assemblers. However, any specific insertion sequences or sites were not determined due to a lack of contiguity, commonly seen in short-read assemblies. Whether the genomic background surrounding a chromosomal insertion site confers or suppresses additional regulatory functions is currently unclear. In the future, long-read sequencing of the *IncQ1* marker-positive LPS-rough isolates should be carried out to elucidate the effect of mobile genetic elements on the gene clusters encoding enzymes for LPS biosynthesis and the development of an LPS-rough phenotype.

Taken together, a more comprehensive characterization of the genomic factors conferring an LPS-rough phenotype could help to improve current tools used for geno-serotyping, which would ensure backward compatibility with slide agglutination-based serotyping, in particular for laboratories that solely use WGS to type isolates. A bioinformatic tool to assess the complexity of genetic factors responsible for the occurrence of LPS-rough *Salmonella* phenotypes as well as their detection in WGS data is currently under development. In addition, elucidating relevant virulence factors and AMR genes could facilitate a more informed and comprehensive assessment of the risk associated with LPS-rough isolates from particular serovars, compared with slide agglutination- or WGS-based serotyping approaches alone, which currently provide an incomplete picture of the relevance of this phenotype for food safety.

### Conclusion

In the present study, we assessed the occurrence and distribution of LPS-rough *Salmonella* isolates in Germany by screening a large data set of *Salmonella* isolates. The data presented here suggest that spontaneous emergence of LPS-rough isolates is the predominant mechanism in the non-human environment and provides no evidence for clonal LPS-rough lineages of *Salmonella* circulating in Germany in general nor in specific environments or production lines. Nevertheless, findings from this national data set suggest that environmental factors associated with certain isolation matrices may favor the development and/or survival of this phenotype, but additional research and focused sampling efforts are needed to corroborate this hypothesis. In addition, while not implying a direct causative association, the overrepresentation of some genomic features in LPS-rough isolates allows for the hypothesis that spontaneously developing LPS-rough isolates that carry certain factors may have a competitive advantage over those that do not. We propose that, due to spontaneous emergence of LPS-rough isolates, animal populations in which an LPS-rough isolate has been detected represent a mixed population of both phenotypic variants. In such cases, extended sampling would reveal LPS-smooth variants, leading to control measures in accordance with European and national legislations. We believe that the data presented here will guide further research into the relevance of this phenotype for food safety and will inform future policy-making and recommendations regarding risk management and control measures.

## Data Availability

All data associated with this work are described in the main text of the manuscript or in Data S 1 and S2. Short-read sequencing data of LPS-rough isolates analyzed in this study have been uploaded to NCBI under BioProjects PRJNA937468, PRJNA1090884, PRJEB31846, and PRJNA742494.

## SUPPLEMENTAL MATERIAL

Data S1 spectrum.02498-25-s0001.xlsx Key information of isolates collected at the NRL for *Salmonella*, including epidemiological metadata, as well as phylogenetic and genomic characteristics.

Data S2 spectrum.02498-25-s0002.xlsx Genomic and metadata features significantly overrepresented in isolates with an LPS-rough phenotype per serovar.

Figure S1 spectrum.02498-25-s0003.pdf Top five sampled isolation matrices per serovar and year among all sampled *Salmonella enterica* subsp. *enterica* isolates (sequenced and un-sequenced).

Figure S2 spectrum.02498-25-s0004.pdf Phylogenetic relationships of sequenced *S*. Infantis and *S.* Enteritidis isolates and associated phenotypic, genomic, and sampling metadata.

Supplemental legends spectrum.02498-25-s0005.pdf Descriptive legends for the supplemental material.

ASM does not own the copyrights to Supplemental Material that may be linked to, or accessed through, an article. The authors have granted ASM a non-exclusive, world-wide license to publish the Supplemental Material files. Please contact the corresponding author directly for reuse.
