# Supplementary figures and images for: Salmonella’s lost phenotype: implications of sequence-based serotyping on the characterization of lipopolysaccharide-deficient Salmonella isolates
Source: Microbiol Spectr. 2026 Jan 27;14(3):e02498-25. doi: 10.1128/spectrum.02498-25 (PMC12955480; doi:10.1128/spectrum.02498-25)

Matrix (top 5 per serovar)

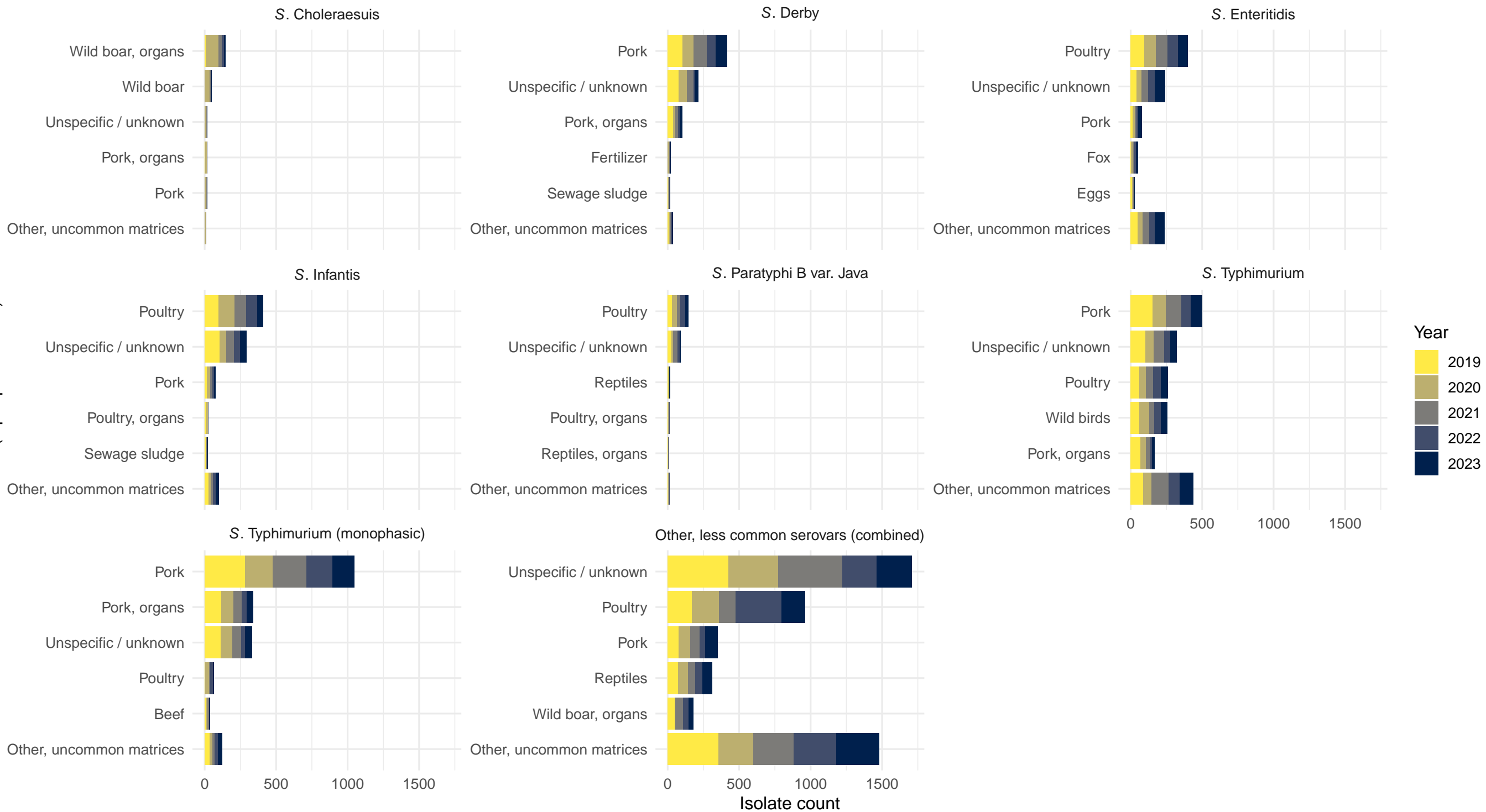

Supplement: Figure S1 — Top five sampled isolation matrices per serovar and year among all sampled Salmonella enterica subsp. enterica isolates (sequenced and un-sequenced). [file spectrum.02498-25-s0003.pdf]

A

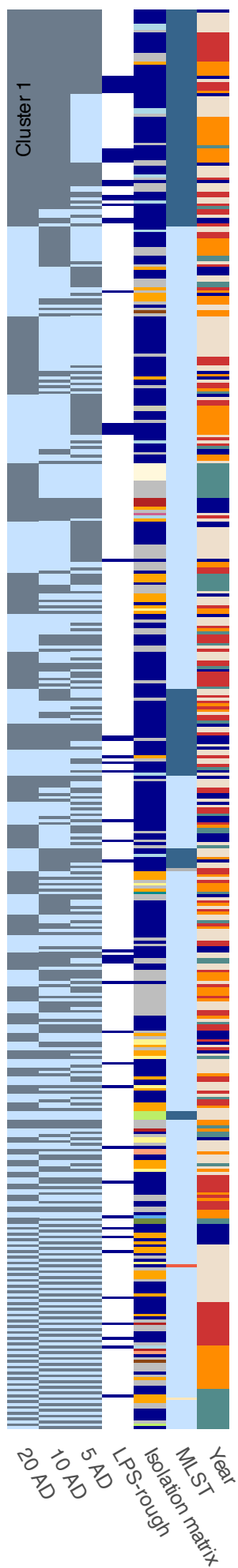

B

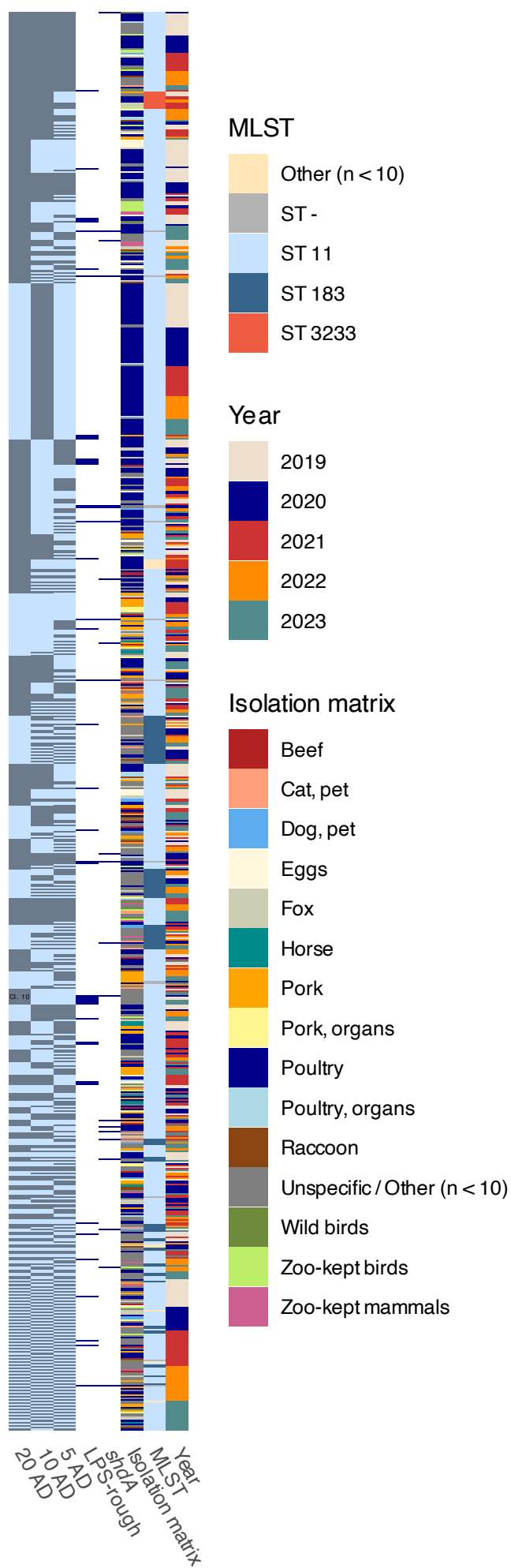

Supplement: Figure S2 — Phylogenetic relationships of sequenced S. Infantis and S. Enteritidis isolates and associated phenotypic, genomic, and sampling metadata. [file spectrum.02498-25-s0004.pdf]
