# Supplementary material for: Salmonella’s lost phenotype: implications of sequence-based serotyping on the characterization of lipopolysaccharide-deficient Salmonella isolates
Source: Microbiol Spectr. 2026 Jan 27;14(3):e02498-25. doi: 10.1128/spectrum.02498-25 (PMC12955480; doi:10.1128/spectrum.02498-25)
Supplement: Supplemental legends — Descriptive legends for the supplemental material. [file spectrum.02498-25-s0005.pdf]

SUPPLEMENTAL MATERIAL

Supplementary Data 1, Key information of isolates collected at the NRL for *Salmonella*, including epidemiological metadata, as well as phylogenetic and genomic characteristics.

Supplementary Data 2, Genomic and metadata features significantly overrepresented in isolates with an LPS-rough phenotype, per serovar.

Supplementary Fig 1 Top five sampled isolation matrices per serovar and year among all sampled *Salmonella enterica* subsp. *enterica* isolates (sequenced and un-sequenced). Uncommon matrices and serovars are collapsed into the category "Other".

Supplementary Fig 2 Phylogenetic relationships of sequenced *S*. Infantis (**A**) (*n* = 491) and *S*. Enteritidis (**B**) isolates (*n* = 888) and associated phenotypic, genomic and sampling metadata. Hierarchical clustering based on core-genome multi-locus sequence typing (cgMLST) is shown for cluster thresholds of 20, 10 and 5 allele distances (ADs) (clusters indicated by alternating gray and light blue blocks). Clusters (20 AD) that showed a statistical overrepresentation of LPS-rough isolates are indicated by name. Among LPS-rough *S*. Enteritidis isolates, the virulence factor *shdA* was overrepresented; its presence/absence is shown for all isolates. In addition, isolation matrix, multi-locus sequence type (MLST) and sampling year are shown for all isolates.
